# Bifidobacterium longum R0175 Protects Rats against d-Galactosamine-Induced Acute Liver Failure

**DOI:** 10.1128/mSphere.00791-19

**Published:** 2020-01-29

**Authors:** Kaicen Wang, Longxian Lv, Ren Yan, Qiangqiang Wang, Huiyong Jiang, Wenrui Wu, Yating Li, Jianzhong Ye, Jingjing Wu, Liya Yang, Xiaoyuan Bian, Xianwan Jiang, Yanmeng Lu, Jiaojiao Xie, Qing Wang, Jian Shen, Lanjuan Li

**Affiliations:** a State Key Laboratory for Diagnosis and Treatment of Infectious Diseases, The First Affiliated Hospital, College of Medicine, Zhejiang University, Hangzhou, China; b National Clinical Research Center for Infectious Diseases, The First Affiliated Hospital, College of Medicine, Zhejiang University, Hangzhou, China; c Collaborative Innovation Center for Diagnosis and Treatment of Infectious Diseases, The First Affiliated Hospital, College of Medicine, Zhejiang University, Hangzhou, China; University of California, Davis

**Keywords:** *Bifidobacterium longum* R0175, acute liver failure, metabolome, microbiome, probiotic

## Abstract

Acute liver failure is a severe liver disorder that poses considerable global challenges. Previous studies on Bifidobacterium longum R0175 have mainly focused on its psychotropic functions. The current research focused on the protective efficacy of B. longum R0175 against acute liver failure caused by d-galactosamine (d-GalN) in rats and further tested the hypothesis that B. longum R0175 exerted liver-protective effects by affecting the intestinal microbiota and fecal metabolites and by inhibiting inflammation. We found that oral gavage of B. longum R0175 markedly reduced the severity of liver injury in d-GalN-treated rats, as evidenced by decreased serum levels of aspartate aminotransferase (AST) and total bile acids (TBAs) (*P* < 0.05). Moreover, the plasma concentrations of proinflammatory cytokines (interleukin 1β [IL-1β] and tumor necrosis factor-α [TNF-α]) and chemokines (granulocyte-macrophage colony-stimulating factor [GM-CSF], macrophage chemoattractant protein 1 [MCP-1], chemokine [C-X-C motif] ligand 1 [CXCL1], chemokine [C-C motif] ligand 5 [CCL5], and macrophage inflammatory protein-1α [MIP-1α]) were also markedly reduced (*P* < 0.05). Pretreatment with B. longum R0175 partially reversed the gut microbiota dysbiosis in rats with liver injury by increasing the relative abundances of potentially beneficial bacteria, such as Alloprevotella spp., and decreasing the relative abundances of potentially harmful bacteria, such as Acetatifactor muris, Butyricimonas spp., and Oscillibacter spp. Furthermore, B. longum R0175 administration partially improved the metabolic function of the intestinal microbes, as indicated by the decreased level of lithocholic acid found in the feces.

**IMPORTANCE** Our research investigated the protective and preventive roles of B. longum R0175 in a rat model of acute liver failure. The results illustrated that this probiotic strain exhibited protective effects in rats with acute liver failure. Thus, B. longum R0175 showed clinical application prospects that required further exploration.

## Abstract (precis)

Our research investigated the protective and preventive roles of B. longum R0175 in a rat model of acute liver failure. The results illustrated that this probiotic strain exhibited protective effects in rats with acute liver failure. Thus, B. longum R0175 showed clinical application prospects that required further exploration.

## INTRODUCTION

Acute liver failure is a severe liver disorder with a 30% mortality rate and presents considerable challenges to clinical management (1). It predominantly arises from viral infections and drug-induced liver injury and is characterized by abrupt hepatic dysfunction, which can lead to hepatic encephalopathy and progressive multiorgan failure (2).

The gut microbiota is the full collection of microorganisms (including bacteria, fungi, viruses, and other microbes) that symbiotically reside in the gastrointestinal tract. The key functions of the microbiota include metabolism, immune regulation, and protection (3) and are closely linked to human health and diseases. Given the bidirectional relationship between the gut and liver in the anatomical and functional context, the gut microbiota is closely associated with different liver diseases. The research revealing that acute exposure to alcohol induced more severe liver injury and inflammation in germfree mice than in wild mice has shed light on the essential protective role of the gut microbiota against liver damage (4). Acute liver failure patients exhibited marked dysbiosis of the gut microbiota, which had a predictive value for mortality (5).

Bacteria of the genus Bifidobacterium are normal inhabitants in the gut and represent a significant part of the healthy microbial community (6). Within the genus *Bifidobacterium*, Bifidobacterium longum is the most abundant species (7). A wide variety of beneficial attributes have been described for this organism, such as activation of immunity (8), participation in metabolism (9), and inhibition of intestinal pathogens (10). B. longum is commonly applied as a probiotic and has been found to hold great promise for protection against liver injury (11–14). *B. longum* R0175 is a strain of B. longum, and until now, most studies concerning this strain have focused on its psychotropic effects (15–17). Thus, whether B. longum R0175 has favorable effects on liver conditions remains unknown.

In the present study, we focused on the protective effects of B. longum R0175 against d-galactosamine (d-GalN)-induced acute liver failure in rats.

## RESULTS

### B. longum R0175 ameliorated d-GalN-induced liver injury.

**FIG 1:**
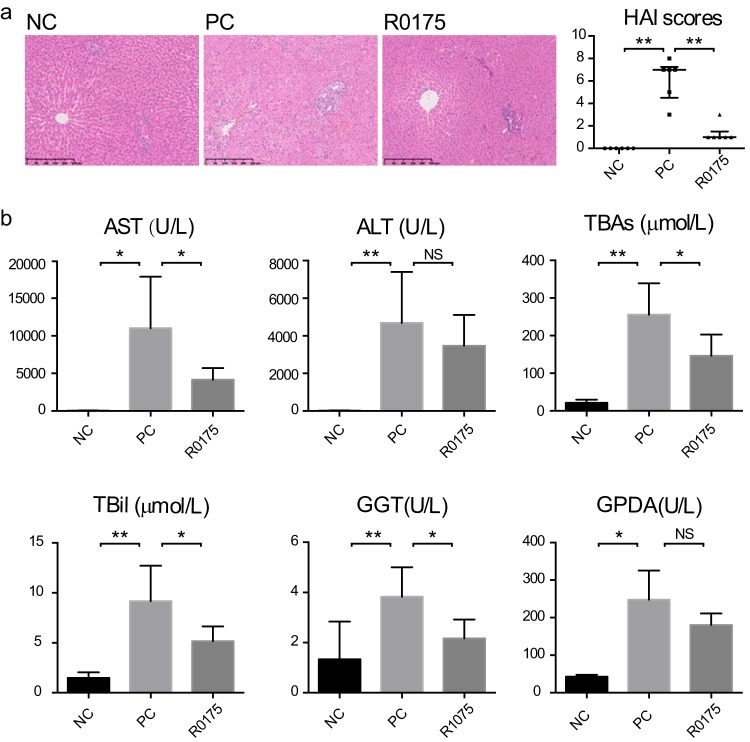
B. longum R0175 administration alleviated d-GalN-induced acute liver injury. (a) Left, representative images of the hepatic histology; right, HAI scores of the liver histopathology. (b) Liver function indexes. HAI scores are given as the median with the interquartile range, and the liver function data are given as the mean ± SEM. Each dot represents one sample (*n* = 6 per group). *, *P* < 0.05; **, *P* < 0.01; ***, *P* < 0.001; NS, no significant difference, compared with the PC group.

Compared with the negative-control (NC; no acute liver failure) group, the positive-control (PC; with acute liver failure) group displayed severe histological liver injury after d-GalN injection, as presented by extensive necrosis, liver tissue destruction, and marked inflammatory cell infiltration, which led to a significant increase in histological activity index (HAI) scores. In comparison with that in the PC group, the d-GalN-induced liver damage in the group orally administered B. longum R0175 was markedly alleviated, which led to decreased HAI scores, as evidenced by reduced necrosis and structural disruption in the hepatic lobules, reduced destruction of the hepatic cell plates, and markedly reduced necrosis and inflammatory cell infiltration in the portal areas of the liver tissue (Fig. 1a).

The liver function test was conducted to evaluate physiological hepatic dysfunction. d-GalN injection sharply increased the serum levels of alanine transaminase (ALT), aspartate aminotransferase (AST), total bile acids (TBAs), gamma-glutamyltransferase (GGT), glycylproline dipeptidyl aminopeptidase (GPDA), and total bilirubin (TBil) in the PC group compared with the NC group. In comparison with the PC group, the B. longum R0175 pretreatment group (R0175 group) had lower concentrations of AST, TBAs, GGT, and TBil (Fig. 1b).

### B. longum R0175 reduced d-GalN-induced intestinal damage.

**FIG 2:**
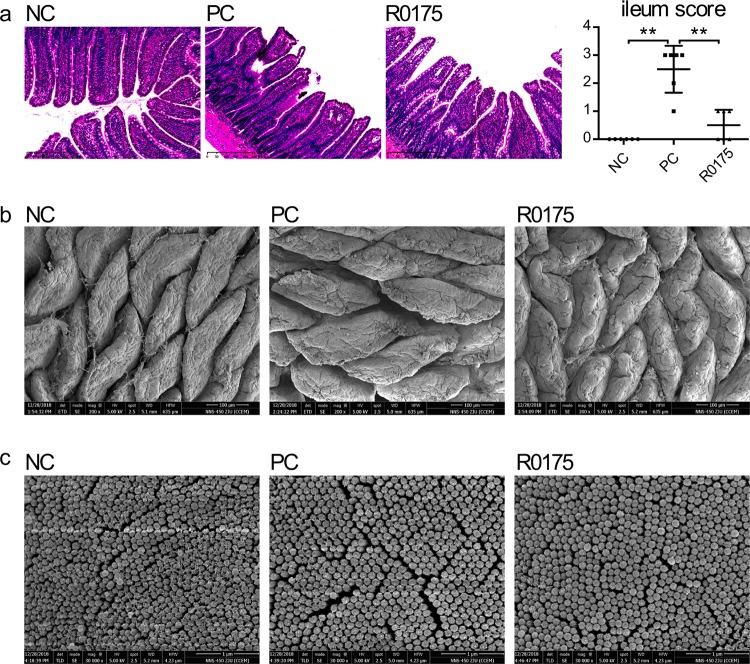
Treatment with B. longum R0175 ameliorated the intestinal mucosal damage. (a) Left, representative images of the terminal ileum histology; right, terminal ileum histopathologic scores. (b) The ultrastructure of the ileal villi observed by SEM. (c) The ultrastructure of the ileal microvilli observed by SEM. Ileum score represents the histological score of the terminal ileum and is given as the median with the interquartile range. Each dot represents one sample (*n* = 6 per group). *, *P* < 0.05; **, *P* < 0.01; ***, *P* < 0.001, compared with the PC group.

The tissue of the terminal ileum was observed under a light microscope to evaluate the intestinal mucosa. Compared with that in the NC group, the integrity of the mucosa in the d-GalN-treated PC group was destroyed with significantly increased intestinal injury scores. However, B. longum R0175 treatment alleviated the d-GalN-induced intestinal mucosal damage. Compared to the PC group, the R0175 group had a lower intestinal injury score, with fewer subepithelial Gruenhagen’s spaces and more intact structures of mucosa and villi (Fig. 2a).

The villi and microvilli of the terminal ileum were further evaluated using a scanning electron microscope (SEM). Compared with the NC group, the PC group displayed shorter and thicker intestinal villi, accompanied by numerous and deeper surface furrows and larger intervillous gaps. Compared to those in the PC group, the villi were rougher, and the spaces between villi were narrower in the R0175 group; however, the villi in the R0175 group were shorter and thicker than those in the NC group (Fig. 2b). Consistent with these findings, the intestinal microvilli were sparser and smoother in the PC group than in the NC group, but pretreatment with B. longum R0175 reduced this disruption (Fig. 2c).

### B. longum R0175 relieved d-GalN-induced systemic inflammation.

The levels of most tested serum inflammatory cytokines were markedly increased in the PC group compared with the NC group. Pretreatment with B. longum R0175 reduced the d-GalN-induced increases in the levels of tumor necrosis factor-α (TNF-α), interleukin 1β (IL-1β), and IL-7 and in chemokines such as granulocyte-macrophage colony-stimulating factor (GM-CSF), chemokine (C-X-C motif) ligand 1 (CXCL1), chemokine (C-C motif) ligand 5 (CCL5), macrophage inflammatory protein-1α (MIP-1α), macrophage chemoattractant protein 1 (MCP-1), and vascular endothelial growth factor (VEGF) (Table 1).

**TABLE 1:** Effects of B. longum R0175 on plasma inflammatory cytokine levels^*a*^

Inflammatory cytokine	Cytokine concn (pg/ml) in group:		
	NC	PC	R0175
TNF-α	16.00 ± 0.00**	105.69 ± 14.39	53.71 ± 20.62*
IFN-γ	8.43 ± 4.43	34.07 ± 10.70	44.81 ± 24.41
IL-1α	4.34 ± 1.14**	19.88 ± 3.23	30.15 ± 8.91
IL-1β	23.19 ± 16.08***	307.29 ± 50.00	60.91 ± 21.95**
IL-2	8.00 ± 0.00**	459.13 ± 95.87	224.42 ± 115.60
IL-4	2.16 ± 1.03*	6.76 ± 1.01	12.06 ± 3.58
IL-5	17.55 ± 11.55**	532.78 ± 300.02	106.41 ± 14.12
IL-6	15.72 ± 2.72**	66.06 ± 24.79	64.10 ± 34.50
IL-7	20.81 ± 15.10**	293.63 ± 62.24	50.93 ± 23.26**
IL-10	5.92 ± 1.92***	33.92 ± 3.44	28.68 ± 5.69
IL-12	7.11 ± 3.11**	22.99 ± 2.27	31.32 ± 13.81
IL-13	2.00 ± 0.00	12.40 ± 8.46	12.82 ± 10.82
IL-17α	2.42 ± 1.42**	9.92 ± 0.77	14.12 ± 2.99
IL-18	182.13 ± 82.33**	746.86 ± 121.57	1158.89 ± 304.78
G-CSF	1.00 ± 0.00	1.07 ± 0.14	1.52 ± 0.35
GM-CSF	25.80 ± 18.45***	260.05 ± 38.53	52.75 ± 24.35**
CXCL1	10.29 ± 5.10***	77.85 ± 4.66	29.80 ± 6.87***
M-CSF	2.28 ± 0.27**	5.17 ± 0.54	6.71 ± 1.15
MCP-1	241.97 ± 45.42***	1120.27 ± 72.96	659.22 ± 46.04***
MIP-1α	6.27 ± 2.86**	342.75 ± 203.86	50.79 ± 11.26**
MIP-3α	3.08 ± 0.40**	11.29 ± 2.03	7.74 ± 0.77
CCL5	34.67 ± 2.36***	74.14 ± 3.82	59.01 ± 6.04*
VEGF	18.64 ± 12.18***	123.83 ± 7.65	51.07 ± 20.87**

a Data are shown as the mean ± SEM; *, *P* < 0.05, **, *P* < 0.01, ***, *P* < 0.001 compared with the PC group.

### Pretreatment with B. longum R0175 alleviated d-GalN-induced gut microbiome dysbiosis.

**FIG 3:**
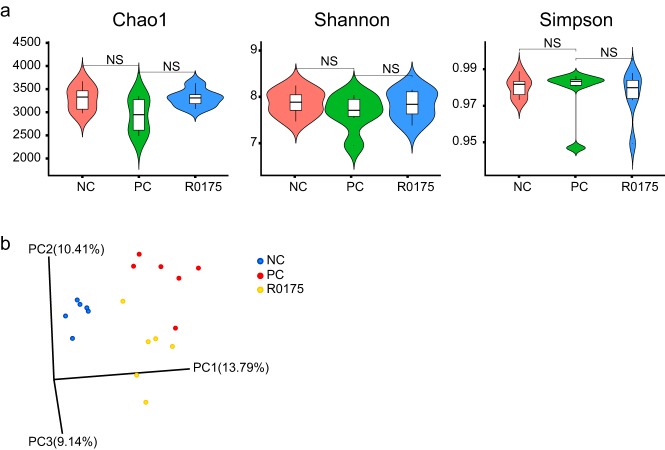
Pretreatment with B. longum R0175 relieved gut microbiome dysbiosis. (a) The violin figures show α-diversity indexes (Chao1, Shannon, and Simpson) of the gut microbiota between the three groups. (b) The PCoA plot shows the β diversity of the gut microbiota between the three groups based on the unweighted UniFrac metric. The α-diversity indexes are given as the median with the interquartile range; NS indicates no significant difference.

16S rRNA sequencing of the fecal pellets was performed to obtain further insights into the impact of B. longum R0175 on the structure of the intestinal microbiota. The α diversity, as represented by the Chao1, Shannon, and Simpson indexes, showed no significant differences between the three groups, indicating that the overall microbial diversity, richness, and evenness were similar among the three groups (Fig. 3a).

Principal-coordinate analysis (PCoA) of the unweighted UniFrac distances was performed to evaluate the β diversity among the three groups. The statistical analysis revealed a distinct separation of the fecal microbiota among the three groups (Fig. 3b). Both the permutational multivariate analysis of variance (PERMANOVA) and the analysis of similarity (ANOSIM) agreed with PCoA in that there were significant differences between microbial communities of these three groups (PC group versus NC group versus R0175 group, PERMANOVA, pseudo-F = 1.98, *P* = 0.001; ANOSIM, *R* = 0.59, *P* = 0.001). Additionally, the microbiota between the two cages in each group was compared using PERMANOVA and ANOSIM, and the results did not show any significant differences between cages (Table 2).

**TABLE 2:** Cage effects on gut microbiota in each group

Group	Data by comparison			
	PERMANOVA		ANOSIM	
	pseudo-F	*P* value	*R*	*P* value
NC-1 vs NC-2	1.35	0.105	0.74	0.107
PC-1 vs PC-2	1.66	0.101	0.91	0.112
R0175-1 vs R0175-2	1.27	0.104	0.44	0.116

**FIG 4:**
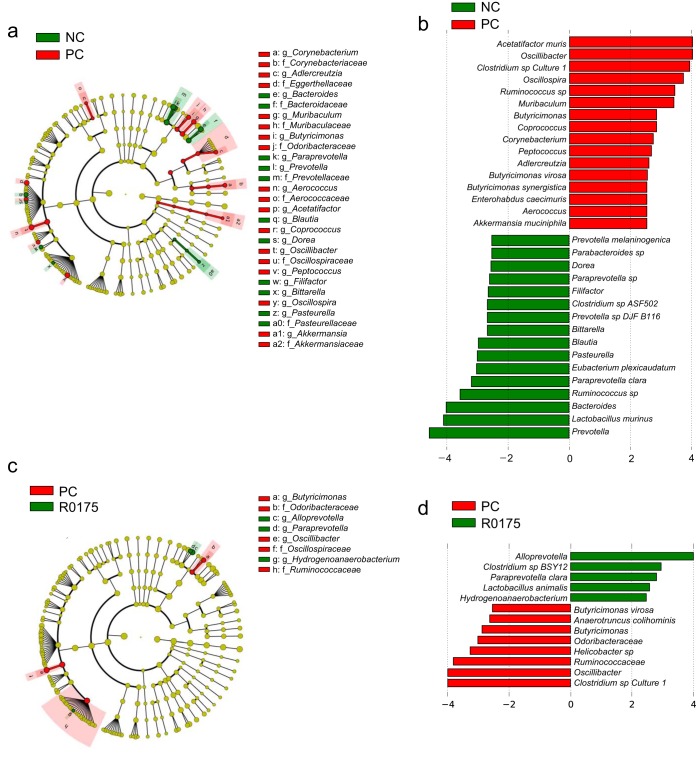
B. longum R0175 administration alleviated microbiome dysbiosis. (a) LEfSe cladograms representing taxa enriched in the NC and PC groups. (b) Discriminative biomarkers with an LDA score of >2.5 or <−2.5 in the PC and NC groups. (c) LEfSe cladograms representing taxa enriched in the PC and R0175 groups. (d) Discriminative biomarkers with an LDA score of >2.5 or <−2.5 in the R0175 and PC groups. Rings in the LEfSe cladograms from the inside out represent taxonomic levels from phylum to genus. The sizes of circles indicate the relative abundances of the taxa.

Linear discriminant analysis (LDA) effect size (LEfSe) analysis at multiple phylogenetic levels was performed to identify differentially microbial biomarkers. The characteristic biomarkers that differed between the NC and PC groups are shown in Fig. 4a and b. The differential microbes between the PC and R0175 groups are shown in Fig. 4c and d. The microbiota in the PC group was enriched with Acetatifactor muris, Akkermansia muciniphila, *Oscillibacter* spp., Oscillospira spp., *Butyricimonas* spp., Butyricimonas virosa, Butyricimonas synergistica, and Clostridium sp. strain Culture_1, whereas it was depleted of Prevotella spp., Bacteroides spp., and Paraprevotella clara compared with those in the NC group. Compared with the PC group, the R0175 group displayed *Oscillibacter* spp., *Butyricimonas* spp., *B. virosa*, and *Clostridium* sp. strain Culture_1 depletion and *Alloprevotella* spp. and *P. clara* enrichment.

### B. longum R0175 ameliorated fecal metabolic profile alterations induced by d-GalN.

**FIG 5:**
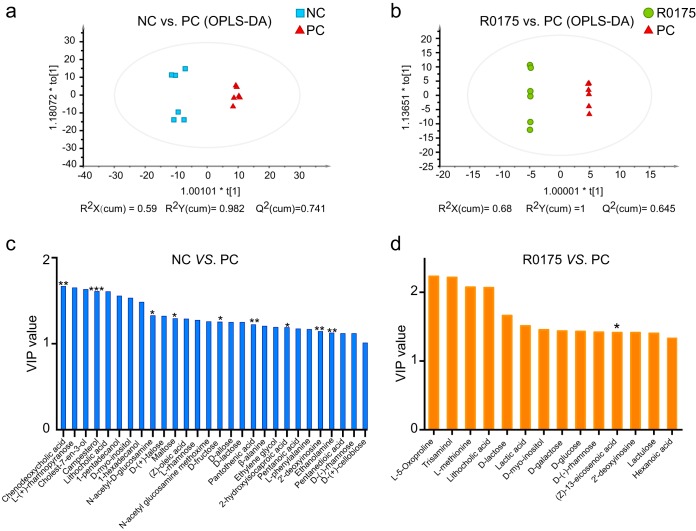
Oral gavage of B. longum R0175 mitigated the changes in the metabolomic profile. (a and b) OPLS-DA plot comparing the NC and PC groups (a) and the PC and R0175 groups (b). Each dot represents one sample. (c and d) The bar charts show metabolites with a VIP value of >1 between the NC and PC groups (c) and between the PC and R0175 groups (d). The asterisk above the bar indicates the *P* value, as follows: *, *P* < 0.05; **, *P* < 0.01; ***, *P* < 0.001. cum, cumulative.

We applied the untargeted gas chromatography-mass spectrometry (GC-MS) analytical method to study the fecal metabolome, which partially reflects the functional features of intestinal microorganisms. Orthogonal partial least-squares discriminant analysis (OPLS-DA) models were established to evaluate the cluster tendencies between the NC and PC groups and between the PC and R0175 groups. Figure 5a depicts the distinct differences in metabolites spectra between the NC and PC groups (*R*^2^*Y* = 0.982, Q^2^ = 0.741), and Fig. 5b shows the metabolite profiling discrimination between the PC and R0175 groups (*R*^2^*Y* = 1, Q^2^ = 0.645). Twenty-eight metabolites with variable importance in the projection (VIP) values of >1 were detected between the NC and PC groups (Fig. 5c), and among these 28 metabolites, campesterol, pantothenic acid, 2′-deoxyinosine, ethanolamine, d-fructose, 2-hydroxyisocaproic acid, and maltose were depleted, whereas chenodeoxycholic acid and *N*-acetyl-d-glucosamine were enriched in the PC group compared with the NC group. Fourteen metabolites with VIP values of >1 were detected between the PC and R0175 groups (Fig. 5d), and among these 14 metabolites, (Z)-13-eicosenoic acid was enriched in the R0175 group compared with the PC group.

**FIG 6:**
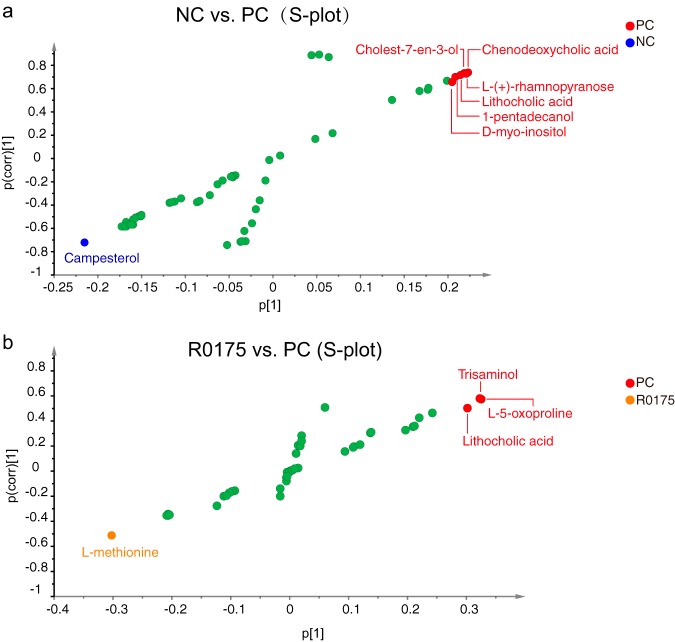
Metabolic biomarkers selected from the S-plot. (a and b) Metabolic biomarkers between the NC and PC groups (a) and between the PC and R0175 groups (b).

Metabolic biomarkers were selected based on the S-plot. Seven metabolites, campesterol, d-*myo*-inositol, 1-pentadecanol, lithocholic acid (LCA), cholest-7-en-3-ol, l-(+)-rhamnopyranose, and chenodeoxycholic acid, had potential value for differentiating the PC group from the NC group (Fig. 6a). These metabolites are associated with pathways such as steroid synthesis, primary and secondary bile acid synthesis, lipid metabolism, l-fucose and l-rhamnose utilization, galactose metabolism, and inositol phosphate metabolism.

Four metabolites, l-methionine, LCA, l-5-oxoproline, and trisaminol, may be potential biomarkers to distinguish the PC group from the R0175 group (Fig. 6b). These four metabolites are mainly involved in pathways such as secondary bile acid biosynthesis, cysteine and methionine metabolism, aminoacyl-tRNA biosynthesis, glucosinolate biosynthesis, and glutathione metabolism.

### Correlations among the differential gut microbes, characteristic metabolites, liver injury indexes, and inflammatory cytokines.

**FIG 7:**
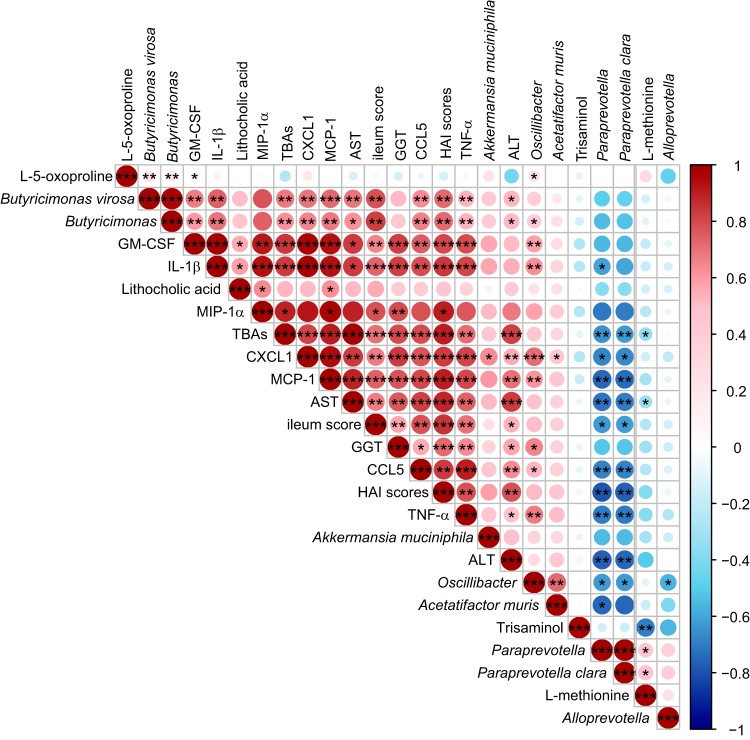
Heat map of the Spearman’s rank correlation analysis of the representative microbes, metabolic biomarkers, liver injury parameters, and inflammatory indexes among the three groups. Color key and circle size indicate the association strength. Dark red indicates a stronger positive correlation, dark blue indicates a stronger negative correlation, and white indicates no correlation. The asterisk in the dot indicates the *P* value, as follows: *, *P* < 0.05; **, *P* < 0.01; ***, *P* < 0.001.

We next performed correlation analyses of the important indexes, including representative microbes, metabolic biomarkers, liver injury parameters, and inflammatory cytokines, among the three groups (Fig. 7). The levels of proinflammatory cytokines (IL-1β and TNF-α) and chemokines (GM-CSF, CXCL1, MIP-1α, CCL5, and MCP-1) were positively associated with the concentrations of ALT, AST, TBAs, and GGT, indicating a widespread positive association between liver injury and systemic inflammation. Certain microbes were closely associated with inflammation and liver damage. The relative abundance of *A. muris* was positively associated with the level of TNF-α. The relative abundances of *Oscillibacter* spp., *Butyricimonas* spp., and *B. virosa* were positively associated with the levels of IL-1β, TNF-α, chemokines (GM-CSF, CXCL1, MIP-1α, CCL5, and MCP-1), and AST and with HAI scores. Notably, the relative abundance of *A. muciniphila* was found to be positively associated with the levels of IL-1β, chemokines (GM-CSF, CXCL1, MIP-1α, and MCP-1), ALT, and AST and with HAI scores. The relative abundance of *P. clara* was negatively correlated with the levels of IL-1β, TNF-α, chemokines (GM-CSF, CXCL1, MIP-1α, CCL5, and MCP-1), ALT, AST, and TBAs and with HAI scores. Changes in the relative abundances of intestinal microbes were accompanied by changes in fecal metabolites. The concentration of LCA was positively correlated with the relative abundance of *B. virosa* and the levels of AST, TBAs, IL-1β, GM-CSF, CXCL1, MIP-1α, and MCP-1. The concentration of l-methionine was positively correlated with the relative abundance of *P. clara* but negatively associated with ALT levels.

## DISCUSSION

Acute liver failure is a life-threatening liver disorder (18). Hepatic failure caused by d-GalN is similar to fulminant viral hepatitis in human (19, 20), and d-GalN-induced acute liver failure models are well characterized and widely used. B. longum is often found in the human gastrointestinal tract and exerts probiotic effects (10). Most studies on B. longum R0175 have focused on its psychobiotic effects on mental illness (15–17). Hence, the current study investigated B. longum R0175-mediated protection against d-GalN-induced acute liver failure. Our main finding showed that pretreatment with B. longum R0175 substantially ameliorated the liver damage resulting from d-GalN injection. Additionally, several beneficial effects of B. longum R0175 were observed, including the attenuation of microbial dysbiosis, improvement of the metabolic profile, and suppression of systemic inflammation.

Serum ALT and AST levels have been known as the major biomarkers for liver injury (21). TBAs engage in various signal transduction pathways, and their levels were elevated following liver injury and hepatic functional changes (22, 23). Compared with other biochemical indicators, the concentrations of TBAs are more valuable for the prognostic evaluation of acute hepatitis (24). In this study, B. longum R0175 treatment distinctly decreased the levels of AST and TBAs in the R0175 group compared with the PC group. The changes in these two functional indexes, along with the improved HAI scores, indicated the alleviation of hepatocyte injury and predicted a better prognosis in the R0175 group than in the PC group.

In addition to pathophysiological damage, acute liver failure was also characterized by the release of inflammatory mediators (25). Upon recognition of a foreign substance, liver Kupffer cells (KCs) are activated, and a wide variety of inflammatory cytokines, including proinflammatory cytokines (e.g., TNF-α, IL-1β, and IL-6) and chemokines (MCP-1, MIP-1α, MIP-1β, and CCL5), are released (26). Importantly, the proinflammatory cytokines TNF-α and IL-1β were considered to make crucial contributions to the pathophysiology and clinical outcomes of severe liver injury (27). The levels of TNF-α and IL-1β were increased after liver injury, and the suppression of these two key inflammatory mediators has been shown to attenuate the liver tissue damage (28). In this study, in the probiotic group, we observed significant reductions in circulating levels of TNF-α and IL-1β, as well as the levels of other important chemokines (GM-CSF, CXCL1, MIP-1α, MCP-1, and CCL5) that recruit inflammatory cells to the liver and accelerate the progression of liver disorders. Notably, we found that the levels of TNF-α, IL-1β, and chemokines were positively associated with the levels of AST, ALT, and TBAs. These results suggest that the inhibition of systemic inflammatory responses may be indispensable for the improvement of liver injury. Furthermore, among the different effects reported for B. longum R0175 is the suppression of inflammation mediated mainly by the reductions in the levels of proinflammatory cytokines, such as TNF-α and IL-1β (29, 30), indicating that B. longum R0175 may prevent the development of acute liver failure by counteracting systemic inflammation.

An altered microbiota has been described in different liver conditions. Thus, we applied fecal microbiome sequencing to identify changes in the microbiota. We found that *A. muris* was enriched in acute liver failure rats, and its relative abundance positively correlated with the level of TNF-α. Recent evidence supported an association of the prevalence of *A. muris* with colitis (31) and obesity (32). We also found a significant increase in the relative abundance of *A. muciniphila* in rats after d-GalN treatment and its positive association with inflammation (IL-1β) and liver damage (AST and ALT). Recently, evidence has been reported supporting the association of enriched *A. muciniphila* with diseases such as type 2 diabetes (33) and multiple sclerosis (34). Hence, it is necessary to further investigate the role and mechanism of *A. muciniphila* in liver diseases. We further found that *Butyricimonas* spp. and *B. virosa* were more abundant in the PC group than in the NC group, and the relative abundances of *Butyricimonas* spp. and *B. virosa* were positively correlated with the concentrations of ALT, AST, IL-1β, TNF-α, and chemokines. Similar results have been observed in other studies. The relative abundance of *Butyricimonas* spp. was increased in hepatocellular carcinoma (HCC) patients with cirrhosis and may be a potential biomarker for HCC (35). Ethanol-induced liver injury increased the relative abundance of *Butyricimonas* spp., which positively correlated with the levels of liver injury parameters such as AST and ALT (36). The species *B. virosa* has been reported to be associated with bacteremia (37–39). We also found that *Oscillibacter* spp. were enriched in rats treated with d-GalN and that their relative abundance was positively associated with the levels of TNF-α, IL-1β, and AST. Various animal experiments have demonstrated that an increase in the relative abundance of *Oscillibacter* spp. played a pivotal role in various chronic metabolic diseases, such as obesity (40), nonalcoholic fatty liver disease (NAFLD) (41), steatohepatitis (42), and type 2 diabetes (43). The elevated relative abundance of *Oscillibacter* spp. correlated with the severity of NAFLD (41). In our study, pretreatment with B. longum R0175 significantly reduced the enrichment of *Butyricimonas* spp., *B. virosa*, and *Oscillibacter* spp., indicating that the shifts in the relative abundances of these microbes may be implicated in the protective effects of the probiotic against acute liver failure in rats. Additionally, we found a positive association between the relative abundance of *Oscillibacter* spp. and intestinal mucosal injury scores, which was consistent with previous studies showing that the enrichment of *Oscillibacter* spp. positively correlated with intestinal mucosal barrier impairment (40, 41). We found that the R0175 group harbored an increased relative abundance of bacteria of the genus *Alloprevotella*, which can indirectly produce short-chain fatty acids (SCFAs). It has been widely known that SCFAs are a major contributor to the maintenance of gut and immune homeostasis (44). The relative abundance of *Alloprevotella* spp. has been reported to be negatively associated with inflammation, insulin resistance, and obesity (45), although we did not find any correlation between the relative abundance of *Alloprevotella* spp., systemic inflammation, and liver injury. Thus, gut microbiota dysbiosis was closely associated with the pathogenesis of d-GalN-induced acute liver failure, and B. longum R0175 supplementation helped relieve the gut dysbiosis and shifted the microbiota to a beneficial profile.

The gut microbiome has previously been described as a virtual metabolic organ (46). Compared to that in the NC group, the fecal metabolic profile in the d-GalN-treated PC group was altered with an increase in the LCA concentration. LCA is exclusively produced by 7-hydroxylation reactions of bacteria in the large intestine, and this conversion is usually performed by a restricted group of bacteria of the order *Clostridiales* (47). The intestinal bile acid profile has been found to be associated with liver injury in animal models (48), and an increase in LCA concentration has been observed in patients with NAFLD (49). Intriguingly, the increase in LCA caused by d-GalN treatment was reduced by B. longum R0175 supplementation. In addition, l-5-oxoproline, which distinguished the PC group from the R0175 group, is an intermediate of the gamma-glutamyl cycle of glutathione synthesis and degradation (50). Previous studies have shown that following the administration of hepatotoxic substances, such as acetaminophen (51) and bromobenzene (52), the l-5-oxoproline concentration increased in different kinds of body fluid or tissue. l-5-Oxoproline has been proposed to be a new and valuable biological marker for the diagnosis of nonalcoholic steatohepatitis (NASH) (53). Notably, our results further showed a negative correlation between the enrichment of fecal l-methionine and liver injury indexes. Methionine is an essential amino acid that participates in the synthesis of protein. Its active form, *S*-adenosyl-l-methionine (SAM), is involved in the proliferation, differentiation, and death of liver cells (54). Reduction in SAM levels was related to progressive liver injury induced by excessive alcohol consumption (55). Given the importance of methionine and its active form (SAM) in the physiology and pathology of the liver, fecal methionine requires further investigation.

As a universal probiotic, B. longum R0175 possesses many beneficial properties and has been widely used in the clinic. This study explored its application in liver diseases and surprisingly found that B. longum R0175 supplementation had a protective effect against acute liver failure in rats. These findings indicated that in addition to its psychotropic prospects, B. longum R0175 has other clinical application prospects that should be explored. However, this study still had many limitations. First, the sample size was not large enough to enable generalization to be made; second, although B. longum R0175 was effective, many preliminary studies are needed to further investigate the functions of this strain.

In summary, B. longum R0175 helped to improve liver injury and to ameliorate the accompanying inflammatory changes in rats with acute liver failure. B. longum R0175 may exert these protective effects by modifying the gut microbiota dysbiosis and the functional profiles.

## MATERIALS AND METHODS

### Strain and culture conditions.

B. longum R0175 was purchased from Lallemand, Inc. (France). The bacteria were cultured on Trypticase-phytone-yeast broth medium (RiShui, Ltd., Qingdao, China) in an anaerobic environment (37°C) for 24 h. The bacteria were harvested by centrifugation (4,000 rpm for 10 min at 4°C) and resuspended at a final concentration of 3 × 10^9^ CFU/ml for further use.

### Experimental procedure.

Male Sprague-Dawley (SD) rats (250 to 350 g) were purchased from Shanghai SLAC Laboratory Animal, Co., Ltd. The rats were raised at room temperature (approximately 25°C) under a 12:12-h light-dark regime with free access to food and water. After a week of acclimatization, the rats were randomly divided into the NC, PC, and R0175 groups (*n* = 6 per group). Every group has two cages (NC-1 and NC-2, PC-1 and PC-2, and R0175-1 and R0175-2), each cage housed three rats together, and the groups were separated by the treatment. The rats in the R0175 group were orally administered 1 ml of B. longum R0175 solution (3 × 10^9^ CFU/ml) per day, while the rats in the PC and NC groups were administered an equal amount (1 ml) of sterile normal saline (NS) for 7 days. On the 8th day, a 1.1-g/kg (of body weight) dose of d-GalN (G0500; Sigma, St. Louis, MO, USA) was injected intraperitoneally to induce acute liver failure in the rats in the PC and R0175 groups, whereas the rats in the NC group received an equivalent dose of NS. The animals were sacrificed after 24 h. Rat feces were collected before sacrifice, and inferior venous blood and tissues (the liver and ileum) were collected for further experiments.

### Liver function tests.

Blood samples from the inferior vena cava were centrifuged (3,000 × *g* for 10 min at 4°C) to segregate the serum or plasma. The serum or plasma was stored at –40°C for further analysis. The concentrations of ALT, AST, GGT, TBAs, TBil, and GPDA in the serum were determined using a 7600 analyzer (Hitachi High-Technologies Corporation, Tokyo, Japan).

### Plasma cytokine analysis.

Plasma cytokine levels were quantified with a Bio-Plex rat cytokine 23-plex assay (Bio-Rad, CA, USA), in accordance with the manufacturer’s protocols. The cytokines that can be evaluated by this kit include TNF-α, gamma interferon (IFN-γ), granulocyte colony-stimulating factor (G-CSF), GM-CSF, CXCL1, CCL5, macrophage colony-stimulating factor (M-CSF), MCP-1, MIPs (MIP-1α and MIP-3α), VEGF, and ILs (IL-1α, IL-1β, IL-2, IL-4, IL-5, IL-6, IL-7, IL-10, IL-12 p70, IL-13, IL-17α, and IL-18).

### Histopathological examination.

The tissues of liver and terminal ileum were collected, soaked in 4% paraformaldehyde solution for fixation, and embedded in paraffin. The paraffin-embedded samples were cut into 2-μm sections, stained with hematoxylin and eosin (H&E), and finally observed under a light microscope. Pathological hepatic tissue damage was evaluated by HAI scoring (56), and pathological changes in intestinal mucosa were assessed as described previously (57).

### SEM.

Terminal ileum specimens were fixed in 2.5% glutaraldehyde (4°C) overnight and then postfixed with 1% OsO_4_ for 1 to 2 h. Then, the samples were dehydrated with a gradient of ethanol solutions (30%, 50%, 70%, 80%, 90%, and 95%) for 15 min each, followed by two cycles of 100% ethanol for 20 min each and dried in a Hitachi model HCP-2 critical point dryer. The dehydrated samples were eventually coated with gold-palladium in a Hitachi model E-1010 ion sputter coater for 4 to 5 min. The coated specimens were observed under a Hitachi model SU-8010 SEM for the structural analysis of intestinal mucosal villi and microvilli.

### Fecal microbiome sequencing.

Fecal bacterial genomic DNA was extracted using a QIAamp fast DNA stool minikit (Qiagen, Hilden, Germany) in accordance with the kit instructions. The total DNA was eluted in 50 μl of nuclease-free water and stored at –80°C until further analysis. Specifically, barcoded universal PCR primers targeting the V3-V4 region of the 16S rRNA gene were used for amplification (338F 5′-ACTCCTACGGGAGGCAGCAG-3′ and 806R 5′-GGACTACHVGGGTWTCTAAT-3′). Following amplification, the products were processed on a MiSeq platform based on the manufacturer’s recommendations (Illumina, San Diego, CA). The raw tags were filtered in the specific filtration context to obtain high-quality clean tags using fqtrim (v0.94). Sequences with ≥97% similarity were assigned to the same operational taxonomic units (OTUs) using Vsearch (v2.3.4). Representative sequences were selected from each OTU, and each representative sequence was given its taxonomic information using the Ribosomal Database Project (RDP) Classifier. Multiple-sequence alignments were conducted to identify the differences in the dominant species in the different groups using the MAFFT software (v7.310) to describe the phylogenetic relationships of the different OTUs. Since the quantity of the fewest sequences of our sample was 63,480 after being filtered, the rarefied OTU data were generated from 1 sequence to 63,480 sequences per sample in steps of 20 by the step size of 3,341 sequences. The α diversity based on normalized OTU data was used to analyze the complexity of the species diversity for the groups. The indexes, including the Chao1, Shannon, and Simpson indexes, were used to represent the β diversity. The rarefaction curves and these indexes were calculated using the software QIIME (v1.9.1). Differences in the species complexities of the samples were evaluated by β-diversity analysis, which was calculated with PCoA and cluster analysis produced by the QIIME software (v1.9.1).

### Fecal metabolomics profiling.

The fecal metabolomics analysis was carried out as described in a previous study (58).

Fifteen milligrams of each fecal sample was mixed thoroughly with 800 μl of methanol (Sigma-Aldrich, St. Louis, MO, USA) and then centrifuged and filtered through a 0.22-μm filter (Millipore, Billerica, MA, USA). The supernatant was transferred to a 1.5-ml tube containing 20 μl of 1 mg/ml heptadecanoic acid (Sigma-Aldrich), which served as the internal standard. The mixture was dried and concentrated with nitrogen (Aosheng, Hangzhou, China). The residue was resuspended in 15 μl of 15 mg/ml methoxyamine pyridine solution (Sigma-Aldrich) and incubated for 24 h (37°C). Then, 50 μl of *N,O*-bis(trimethylsilyl)trifluoroacetamide (BSTFA) with 1% trimethylchlorosilane (TMCS; Sigma-Aldrich) was added, and the solution was incubated again for 2 h (70°C) for derivatization. The products were subjected to metabolomics analysis using GC-MS on a 7890A GC system coupled to a 5975C inert mass selective detector (MSD) system (Agilent Technologies, Santa Clara, CA, USA).

The data were analyzed using Qualitative Analysis B.07.00 (Agilent, Santa Clara, CA, USA). Metabolites were identified using the NIST 17 database. The metabolic clustering between groups was evaluated by OPLS-DA. A VIP value in the OPLS-DA model of >1 was taken as the standard criterion to measure the influence of metabolites for sample classification. Metabolic biomarkers between groups were chosen according to the S-plot of the OPLS-DA based on |*P*(1)| of >0.2 and |*P*(corr)| of >0.5. The KEGG database and the Human Metabolome Database (HMDB) were used to search for metabolic pathways associated with the characteristic metabolites.

### Ethics statement.

All procedures were performed according to the 2011 National Institutes of Health Guide for the Care and Use of Laboratory Animals and were approved by the Animal Care and Use Committee of the First Affiliated Hospital, School of Medicine, Zhejiang University.

### Statistics.

Whether the data satisfied the normal distribution criteria was determined by the Kolmogorov-Smirnov test. If satisfied, ANOVA followed by *post hoc* least significant difference (LSD) testing was used to analyze the significant differences between groups, and if not, a nonparametric test (Wilcoxon rank sum test) was applied to analyze the significant differences between groups. PERMANOVA and ANOSIM based on the unweighted UniFrac distance metrics were applied to determine the clustering of the microbial communities. LEfSe (http://huttenhower.sph.harvard.edu/galaxy/) was used to identify the taxa that explain the differences in microbial communities between the NC and PC groups and between the PC and R0175 groups (59). Taxa with an LDA score of >2.5 or <−2.5 and a *P* value of <0.05 were considered to be significant in our study. Correlations between metabolites, bacteria, immune function markers, and liver function indexes were calculated using Spearman’s rho. If the data satisfied the normal distribution criteria, they were presented as the mean ± standard error of the mean (SEM); if not, they were shown as median with interquartile ranges. The *P* value was adjusted using the Benjamini-Hochberg method, and the criterion for a significant difference was set to <0.05. SPSS version 20.0 was used for data analyses (SPSS, Inc., Chicago, IL, USA).

### Data availability.

All data generated or analyzed during this study are included in this published article. The data sets generated during the current study are available in the GenBank Sequence Read Archive repository under BioProject number PRJNA575606.
